# Supplementary material for: Anti-Inflammatory and Immunomodulatory Effects of 0.1 Sub-Terahertz Irradiation in Collagen-Induced Arthritis Mice
Source: Int J Mol Sci. 2024 May 29;25(11):5963. doi: 10.3390/ijms25115963 (PMC11172739; doi:10.3390/ijms25115963)
Supplement: Supplementary file 1 [file ijms-25-05963-s001.zip › 3-supplement information .pdf]

Anti-Inflammatory and Immunomodulatory Effects of 0.1 Sub-Terahertz Irradiation in Collagen-Induced Arthritis Mice

Qi Zhang, Sen Shang, Xu Li and Xiaoyun Lu *

Key Laboratory of Biomedical Information Engineering of the Ministry of Education, School of Life Science and Technology, Xi'an Jiaotong University, Xi'an 710049, China; qqqizhang609408@126.com (Q.Z.); shangsen2106@xjtu.edu.cn (S.S.); lixu15668451108@163.com (X.L.)
* Correspondence: luxy05@xjtu.edu.cn; Tel: +86-15353713886

Supporting Information

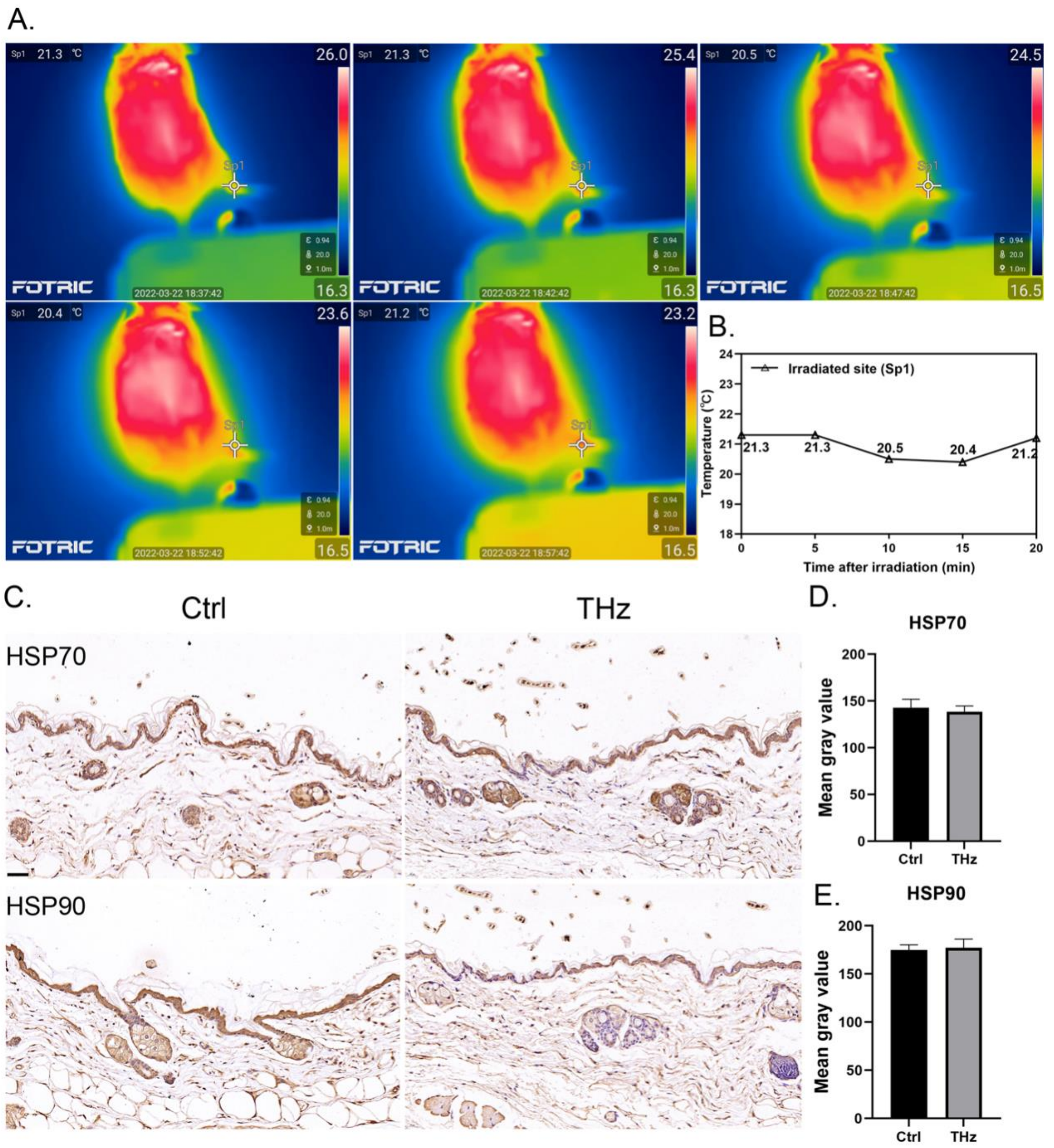

Supplement Figure S1. Mainly a non-thermal effect under this parameter of THz irradiation source. A. Real time temperature of irradiation site. B. Temperature change curve during THz irradiation. C. IHC staining of HSP70 and HSP90 in skin of irradiated area (THz group) or corresponding normal skin (Normal group), respectively. Scale bar: 100 μm. D. Mean gray value of HSP70. E. Mean gray value of HSP90. Data are presented as the mean ± SEM (n = 3 mice per group).

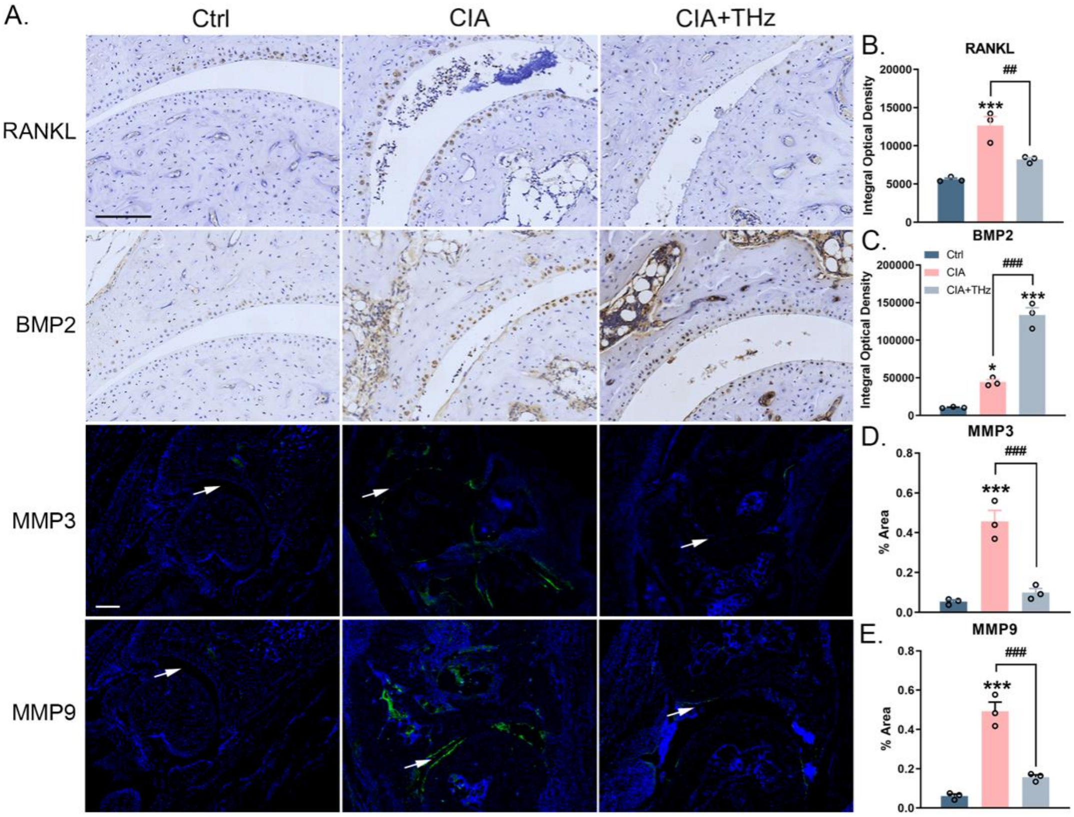

Supplement Figure S2. IHC or IF staining of RANKL, BMP2 and Matrix metalloproteinase (MMPs) of ankle joint of mice. A. IHC or IF staining of RANKL, BMP2, MMP3 (green), MMP9 (green), and DAPI (blue) in ankle joint from Ctrl, CIA and CIA+THz group, respectively, and representative areas are displayed. B. Integral optical density of IHC staining of RANKL (***P < 0.001 and $^{\#\#}P < 0.01$). C. Integral optical density of IHC staining of BMP2 (*P < 0.05, ***P < 0.001 and $^{\#\#\#}P < 0.001$). D. Percentages of positive area of inflorescence staining of MMP3 (***P < 0.001 and $^{\#\#\#}P < 0.001$). E. Percentages of positive area of inflorescence staining of MMP9 (***P < 0.001 and $^{\#\#\#}P < 0.001$). D. Percentages of positive area of inflorescence staining of MMP13. Data are presented as the mean ± SEM (n = 3 mice per group). White arrows: Joint cavity; Scale bar: 100 μm. Symbol for the significance of differences compared to the Ctrl group: ***P < 0.001; Symbol for the significance of differences between the CIA group and CIA+THz group: $^{\#\#\#}P < 0.001$.

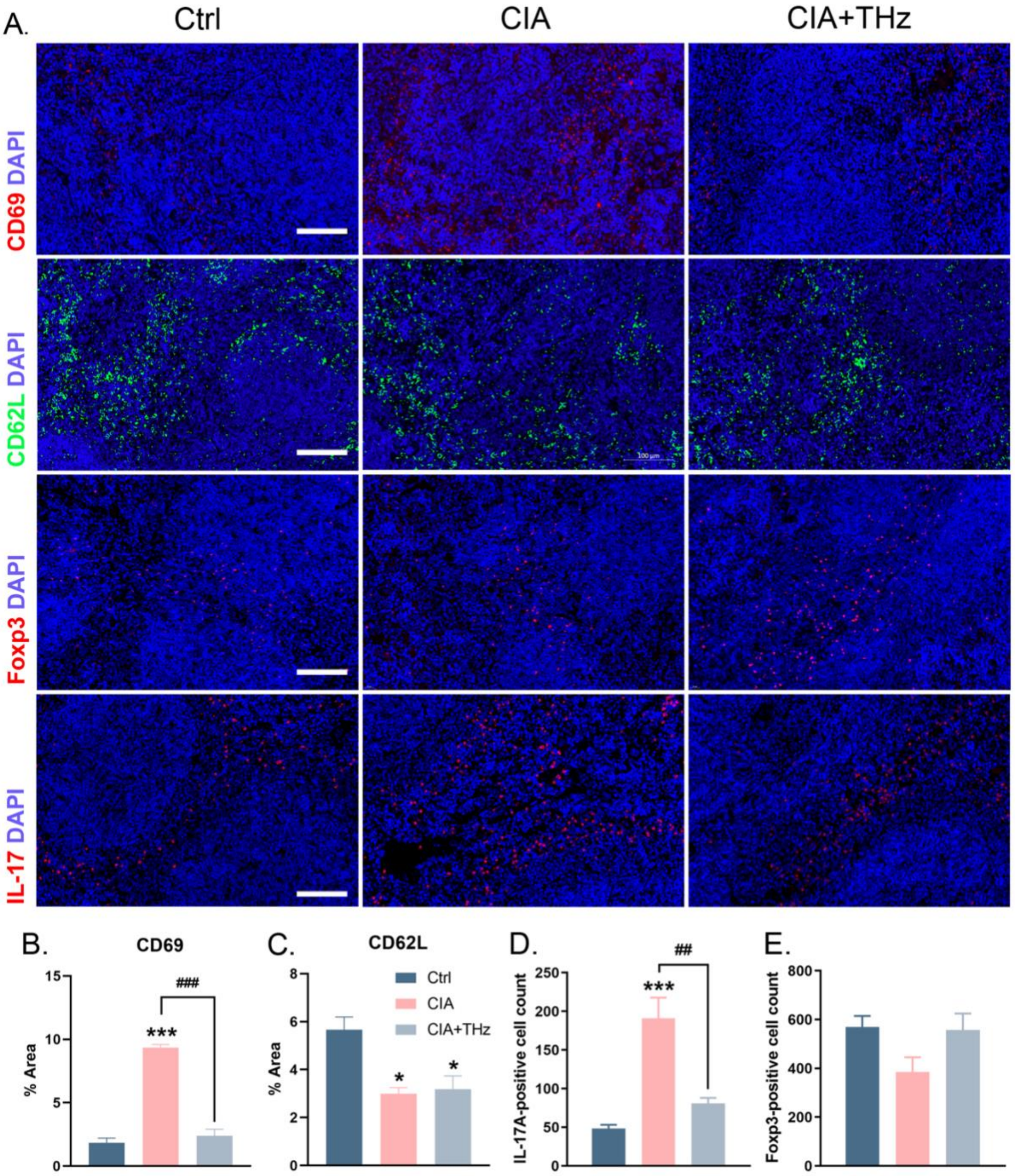

Supplement Figure S3. IF staining of T cell surface marker of mice spleen. A. IF staining of CD69 (red), CD62L (green), Foxp3 (red), IL-17 (red) and DAPI (blue) in mice spleen and representative areas are displayed. Scale bar: 100 μm. B. Percentages of positive area of inflorescence staining of CD69 (****P* < 0.001 and ###*P* < 0.001). C. Percentages of positive area of inflorescence staining of CD62L (**P* < 0.05). D. Percentages of positive area of inflorescence staining of IL-17 (****P* < 0.001 and ##*P* < 0.01). E. Percentages of positive area of inflorescence staining of Foxp3. Data are

presented as the mean ± SEM (n = 3 mice per group). Symbol for the significance of differences compared to the Ctrl group: *P < 0.05 and ***P < 0.001; Symbol for the significance of differences between the CIA group and CIA+THz group: $^{\#\#}P < 0.01$ and $^{\#\#\#}P < 0.001$.

Supplement table S1. mRNA Anova Gene Differential Expression Summary.

Supplement table S2. mRNA Anova Gene GO Enrichment Summary

Supplement table S3. mRNA Anova Gene KEGG Enrichment Summary

Supplement table S4. Gene Differential Expression Summary of |log2FC|> 0.67

Supplement table S5. GO enrichment of |log2FC|> 0.67 of DEGs
